# A New Departure in the Medical Expert Matter

**Published:** 1912-05

**Authors:** S. W. Little

**Affiliations:** Rochester, N. Y.


### A New Departure in the Medical Expert Matter.

DR. S. W. LITTLE
Rochester, N. Y.

WE of the Monroe County Society, being convinced that attempts at the regulation of medical expert testimony have failed and must fail for some time to come for various reasons, political, business and temperamental, have decided to attack the problem in a new way. We believe that the united medical profession can force a reform in this matter without legal, financial or any other aid. We believe that our State Society could do it but won't—yet. We think that in Monroe County the County Society can do it and we are going to try. Our hope is that we shall succeed and if we do, our confident expectation is that other county societies will follow suit and eventually the State Society. If New York State, then other states.—An ambitious program, but miracles are happening all the time.

Here is our scheme which was formally adopted by the society at its recent regular meeting: At the next regular meeting the censors will recommend to the society for adoption a list of experts in Insanity. (We start with Insanity alone—wisely, our joking critics say).

The Society will then vote on these names and any one receiving a three-fourths vote of members present will be vouched for by the Society as an expert in Insanity for one year.

Anyone desiring to go on the list may at any time present his name and credentials to the censors exactly as for admission to membership in the Society. It is the plan to be very liberal in the matter of certification, the object being of course, to prevent grossly incompetent men from posing as experts.

The list of names properly certified will then be sent with an explanatory note to every lawyer and judge practicing in this County. That is all.

No one will be obliged to employ these certified experts but it is reasonable to suppose that they will be preferred whenever possible, because an expert vouched for by the profession would have more weight than one qualified by his own say-so alone.

The possible results of such a scheme are interesting to speculate upon but in another year we shall have more than speculation to report. If good seems to follow, our plan is to present in like manner lists of experts in other lines of medicine and surgery.

There is at least one desirable feature that is sure to result. The Monroe County Medical Society will be in a dignified position as regards expert medical testimony, a position not held at present by any medical society or by any medical man in this country. We say in effect to court, lawyers and public: "Here is a list of medical men whom we, their associates, consider experts in Insanity. You may use them or not as you see fit, but if you do not use them you cannot blame the medical profession for any more medico-legal scandals."

S. W. LITTLE,
President, Medical Society of the Co. of Monroe.

